# Experimental evaluation of hard hat performance during impacts to a forward-flexed head

**DOI:** 10.1080/23335432.2025.2560410

**Published:** 2025-09-17

**Authors:** Arthur Santos, James Sorce, Mohammadhadi Masoudi, Alexandra Schonning, Grant Bevill

**Affiliations:** a Department of Engineering, University of North Florida, Jacksonville, FL, USA; b Department of Construction Management, University of North Florida, Jacksonville, FL, USA

**Keywords:** Construction safety, personal protective equipment, Hybrid III, traumatic brain injury, head accelerations

## Abstract

Traumatic brain injuries frequently occur in the construction industry when workers are struck with their heads in a forward-flexed position. Hard hats, the primary form of protection against such events, may not be optimized for these forward-flexed impacts since certification testing is performed with a head form in an upright configuration. In response, this study assesses the impact mitigation of six commercially available hard hats when subjected to impacts in different head orientations – upright and with 30° of forward flexion. Impactors with a mass of 3.6 kg were dropped vertically onto a Hybrid III 50^th^ percentile head/neck form. Kinematic outcomes related to TBI (e.g. accelerations) were compared across impact conditions. Results indicated that impacts to the forward-flexed head resulted in the largest angular accelerations, and hard hats were the least effective at mitigating angular accelerations in this head position. Furthermore, correlation analysis indicated that hard hats that performed well in upright testing often performed poorly in forward-flexed conditions (or vice versa). Taken together, these results suggest consumers/employers are not equipped with the necessary information to select the safest products for all impact conditions based on safety certification results using upright testing alone.

## 1. Introduction

Construction and industrial work are widely recognized as some of the most dangerous occupational sectors, with high rates of injury and fatality observed both in the United States and internationally (Konda et al. 2016; CPWR 2018; Bottlang et al. 2022; Sanjeewani et al. 2024; Kiral and Demirkesen 2024). A significant proportion of these injuries results from struck-by incidents, including those involving falling tools, equipment or building materials (Janicak 1998; Liu et al. 2011; Konda et al. 2015, 2016). These events are a leading cause of traumatic brain injuries (TBIs) among construction workers worldwide (Hulme et al. 1995; Kim et al. 2006; Kristman et al. 2008; Konda et al. 2015, 2016; Chang et al. 2015). For example, Colantonio et al. reported an incidence rate of mild TBIs of 49 cases per 10,000 full-time construction employees in Canada (Colantonio et al. 2009), while similar findings have been reported in studies from the United Kingdom, Australia and South Korea (Kim et al. 2016; Safe Work Australia 2022; HSE 2024). As the conventional means of head protection in construction, hard hats are critical in mitigating the severity of these injuries. However, it is not clear that current helmet designs are optimized to prevent TBI or that they provide consistent protection across all real-world impact configurations.

Hard hats are certified using standardized testing procedures that evaluate performance with a vertically oriented impactor striking a head form in a neutral, upright position (American national standard for industrial head protection 2014). However, data from incident investigations and observational studies suggest that many injurious impacts occur when the head is not upright – particularly in forward-flexed postures common during tasks such as lifting, bending or inspecting work (1985). This raises concerns about the relevance of certification test results for predicting protection during non-upright head orientations. From a biomechanical perspective, the forward-flexed posture may increase the risk of injury by altering the kinematics of the head, particularly by increasing rotational acceleration. This is important because mild TBIs are more strongly associated with rotational kinematics than linear (Schmitt 2010; Kleiven 2013).

Although prior studies have examined the performance of various helmet types, including traditional hard hats, climbing-style helmets and helmets with rotation-damping technologies (Bottlang et al. 2022), little is known about how these devices perform when the head is in a forward-flexed position when impacted by a falling object. To our knowledge, only one previous study has evaluated hard hat response to non-vertical loading (which specifically investigated lateral loading (Thomson et al. 1997)), and no data currently exist for vertical impacts to a forward-flexed head. As such, understanding helmet performance during impacts to a forward-flexed posture is critical for evaluating injury risk and identifying limitations in current personal protective equipment (PPE). The present study addresses this gap by comparing the impact response of hard hats during vertically oriented impacts to both upright and forward-flexed head postures.

Within this context, this study seeks to evaluate protective capabilities of hard hats for forward flexed versus upright head postures, as assessed by common biomechanical injury metrics, including peak linear acceleration (PLA), peak angular acceleration (PAA) and head injury criterion (HIC). Second, this study seeks to evaluate whether the impact performance of hard hats tested in an upright head posture is predictive of performance in forward-flexed impacts. Such data could be beneficial for understanding occupational injury risk and for beginning the process of optimizing hard hats for a variety of the most common impact configurations.

## 2. Materials and methods

**Figure 1.**
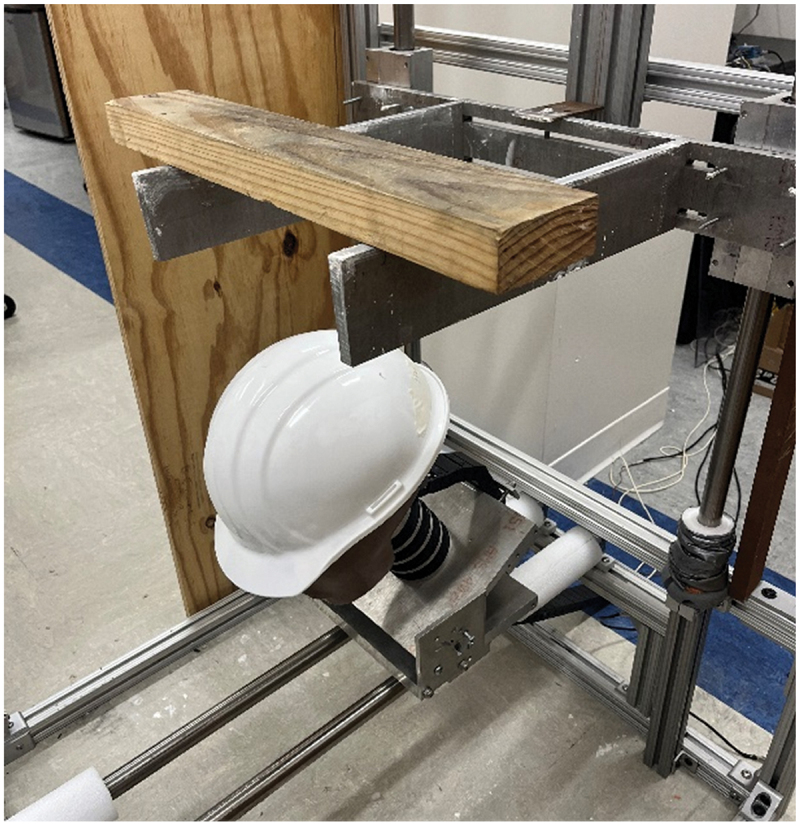
Photograph of the drop rig fixture used in experiments, showing the head in a forward-flexed position prior to impact.

The experimental test fixture used consisted of an extruded aluminum frame, with two linear rail systems. A set of aluminum plates were connected to the vertical rails via low-friction pillow block bearings to bear the impactor and allow the object to be positioned in line with the center of gravity of the head form. The anthropomorphic test device (ATD) – a Hybrid III 50^th^ percentile male head/neck form – was mounted at the bottom of the frame, where it was rigidly attached to another set of aluminum plates (Figure 1). This group of plates had the ability to be fixed at any angle ranging from 0° to 90°. These plates were attached to a horizontal linear rail system via low-friction pillow blocks, enabling the translation of the ATD in the anterior-posterior direction after the impacts. Similar testing format and structure has been used and reported elsewhere in literature (American national standard for industrial head protection 2014; Post et al. 2015).

### 2.1 Testing parameters

Three impactor types were used to conduct the tests: steel, wood and lead shot. Steel and wood objects are the first and second most common source of injury for head injuries, respectively (1985). The third type of impactor chosen was lead shot, as it can simulate collections of loose metallic items, such as nails, screws or nuts. All impactors were fabricated to have a weight of 3.6 kg, which was chosen since it matches the standard used in current hard hat testing by ANSI Z89.1 2014 (American national standard for industrial head protection 2014). The impactors were positioned 1.83 m above the ATD head form, consistent with the range of the most frequent impact heights according to BLS (1985).

The base plate of the test fixture can be locked in a variety of angled positions to simulate forward flexion of the head. Two head position configurations were selected for this study, upright (also called neutral) and forward flexed. The angle of flexion used for the test configuration was 30°, chosen based on data collected for the work tasks and associated body postures in the construction industry (1985; Moriguchi et al. 2013). For all tests (upright and forward flexed), the impactors were aligned with the center of gravity of the head form.

### 2.2. Equipment description

**Figure 2.**
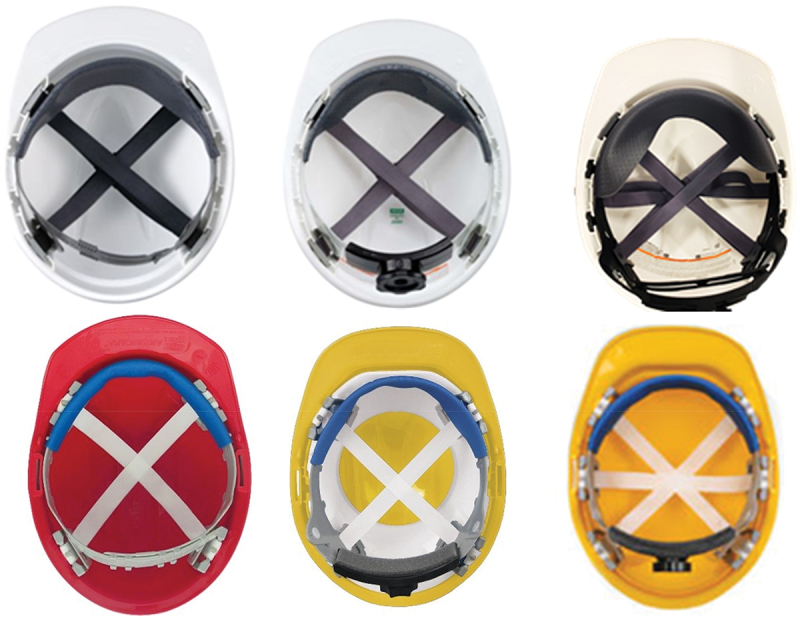
MSA (top) & ERB (bottom) hard hats: 4-point pin-lock (left), 4-point ratchet (middle) and 6-ratchet (right) designs were tested from each manufacturer (MSA safety 2019; ERB safety^2019^).

The hard hats selected for this experiment were six models from two different brands with three distinct suspension systems, as seen in Figure 2. The different designs represent the most common commercially available construction head PPE regarding styles of strap and suspension systems. Each hard hat was tested three times for each combination of impactor (steel, wood and lead shot) and head position (upright (0°) and forward flexed (30°)). Additionally, tests without helmets (unprotected) were also conducted for each test condition, resulting in a total of *N* = 126 tests.

### 2.3. Data analysis

Data were collected via a data acquisition system (Slice Micro, DTS, Seal Beach, CA) mounted at the CG of the headform, which was set to sample acceleration data at 20 kHz with 4 kHz anti-alias filtering for three linear accelerometers and three angular rate sensors (DTS 6DX PRO 2K-18K, Seal Beach, CA). The data were then post-processed through a custom MATLAB program (The MathWorks, Inc., Natick, MA) in accordance with SAE J211 (Instrumentation for Impact Tests) using a channel frequency class of 1000; this standard specifies that phaseless (zero-phase) filtering be applied via a 4th-order Butterworth low-pass filter (forward and backward). Angular accelerations and other related injury metrics were calculated in accordance with SAE J1727 (Calculation Guidelines for Impact Testing). The injury criteria used in this analysis and the results from the post-processed data are expressed in terms of peak linear acceleration (PLA), peak angular acceleration (PAA) and Head Injury Criterion (HIC).

Analysis of variance (ANOVA) was performed using SPSS (IBM, Armonk, NY) to assess factors influencing magnitudes of measured injury criteria, thereby providing insight to the first objective of this study – to evaluate the basic biomechanical response for upright versus forward flexed impacts. Two sets of this statistical tool were generated. First, three 4-way ANOVAs were conducted, where the independent factors were: hard hat design, impactor material, head orientation and the dependent factor for each ANOVA was an injury metric (e.g. PLA, PAA or HIC). The second set consisted of three 3-way ANOVAs, where orientation was not a factor, therefore exclusively comparing the angled impacts among themselves. Tukey HSD post hoc tests were performed on any ANOVA where overall significance was detected to investigate statistical differences across each factor and their interaction.

To investigate whether the performance of hard hats in upright testing were predictive of forward-flexed results, correlation analysis was performed using two methods – Pearson’s linear correlation and Spearman’s rank correlation. Specifically, Spearman’s rank correlation was used for evaluating the consistency of performance rankings between conditions. A Pearson’s correlation was also performed since rank-based methods do not capture information about magnitude differences in performance (such as if two hard hats differ by only a small margin in acceleration but receive different ranks in the Spearman correlation). Additionally, scatter plots – separated by impact condition and injury metric (e.g. 3.6 kg steel bar – PLA 0° X PLA 30°) – were created to depict the relationship between outcome metrics measured in vertical versus forward flexed impacts.

This research was conducted without any patient and public involvement. There was no involvement in the study design, procedures, analysis of results by patients or public.

## 3. Results

**Figure 3.**
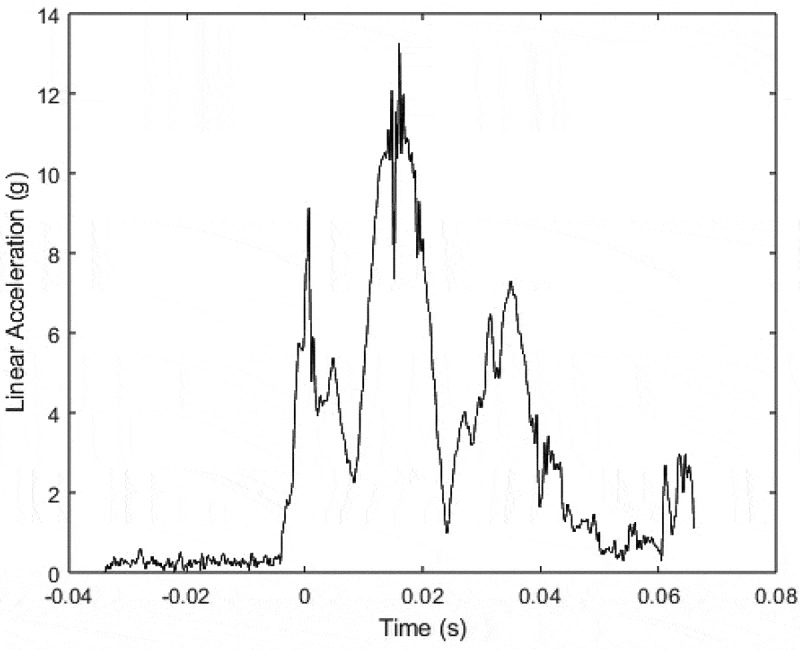
Resultant linear acceleration versus time for a representative impact (MSA 6-point strap with ratcheting mechanism, impacted by eight-pound lead shot). Note that the resultant is calculated as the vector norm of the three orthogonal components of linear acceleration measured in the head form. The peak acceleration for this particular test was 13.2 g’s.

A total of *n* = 126 impact tests were conducted and analyzed. A representative acceleration curve showing the resultant linear acceleration (vector norm of the three linear components) versus time for a single test is shown in Figure 3. For impacts to an unprotected head form, the upright head position tended to result in larger values for PLA and HIC (reaching significant statistical differences, *p* < 0.001, for PLA and for HIC for any impactor) whereas the forward flexed position produced significantly greater values of PAA (*p* < 0.001). These trends did not remain the same when the hard hats were added (Table 1). Specifically, most linear outcome metrics (PLA and HIC) were significantly higher when the head was forward-flexed (*p* < 0.05). Angular metrics consistently remained higher for the forward flexed position even with the use of the hard hats (*p* < 0.05) – the highest values measured in upright head testing were less than the lowest values measured during forward-flexed testing. The hard hats were not effective at reducing PAA across any test conditions with the forward-flexed head posture. For example, for the lead shot impactor, the best performing hard hat reduced PAA by only 25% and the worst performing hard hat was equivalent to the unprotected condition (*p* > 0.90).

**Table 1.** Testing outcomes for upright versus forward-flexed head positions, including unprotected and range of hard hat outcomes (range was selected as the performer with the smallest mean and largest mean, with ±2 standard deviations reported; the selection of a low and high end of the range does not necessarily imply significant differences).

		Upright		Forward-Flexed	
		Unprotected	Hardhat Range	Unprotected	Hardhat Range
Steel	PLA (g’s)	331.0 ± 3.4	27.9 ± 7.4 to 33.7 ± 2.2	222.6 ± 11.1	31.7 ± 1.5 to 41.55 ± 4.79
	HIC	720.8 ± 19.2	14.5 ± 4.8 to 20.22 ± 5.11	290.6 ± 6.6	16.2 ± 1.2 to 30.3 ± 2.0
	PAA (rad/s^2^)	2751 ± 836	1279 ± 63 to 1694 ± 161	4225 ± 153	2012 ± 98 to 3117 ± 728
Wood	PLA (g’s)	247.1 ± 6.9	28.7 ± 1.4 to 38.6 ± 3.4	221.0 ± 6.2	28.7 ± 0.9 to 39.6 ± 0.7
	HIC	472.1 ± 11.5	15.9 ± 1.2 to 20.7 ± 2.9	417.6 ± 16.6	16.2 ± 1.2 to 22.6 ± 0.9
	PAA (rad/s^2^)	2608 ± 576	1313 ± 24 to 1509 ± 72	8529 ± 760	1608 ± 106 to 2393 ± 105
Lead Shot	PLA (g’s)	74.5 ± 11.3	12.7 ± 0.7 to 15.1 ± 1.0	67.3 ± 14.6	13.0 ± 1.5 to 16.5 ± 0.3
	HIC	25.4 ± 8.2	2.8 ± 0.4 to 4.0 ± 0.6	17.5 ± 8.9	3.1 ± 0.2 to 5.0 ± 0.7
	PAA (rad/s^2^)	885 ± 148	605 ± 124 to 697 ± 115	1431 ± 152	1071 ± 104 to 1445 ± 150

After adjusting for all factors, the ANOVA indicated that PLA, PAA and HIC were significantly higher for impacts in a forward-flexed posture as compared to upright (*p* < 0.001). This was further confirmed via orientation pairwise comparisons for all injury metrics were (*p* < 0.001). Tables 2, 3 – 4 display all the test results averaged for each hard hat separated by impactor type.

**Table 2.** Steel impactor test results for PLA, PAA and HIC for upright (U) and forward-flexed (F) head positions.

PPE	Head Orientation	PLA (g)	PAA (rad/s^2^)	HIC
MSA 4 Pin	F	41.4 ± 3.1	3001 ± 846	30.3 ± 2.0
	U	27.9 ± 7.4	1324 ± 288	14.5 ± 4.8
MSA 4 Ratchet	F	41.6 ± 4.8	3117 ± 728	26.9 ± 2.8
	U	29.6 ± 1.7	1279 ± 63	15.1 ± 5.0
MSA 6 Ratchet	F	38.6 ± 1.9	2657 ± 218	24.8 ± 1.2
	U	32.9 ± 3.3	1694 ± 161	20.2 ± 5.1
ERB 4 Pin	F	33.9 ± 2.7	2158 ± 186	16.2 ± 1.2
	U	30.3 ± 0.4	1389 ± 228	18.8 ± 1.7
ERB 4 Ratchet	F	31.7 ± 1.5	2012 ± 98	17.9 ± 0.4
	U	33.7 ± 2.2	1371 ± 75	18.2 ± 1.9
ERB 6 Ratchet	F	32.8 ± 2.4	2095 ± 155	19.7 ± 0.9
	U	28.92 ± 1.13	1347 ± 84	17.6 ± 0.2
Unprotected	F	237.8 ± 21.1	18748 ± 13580	334.1 ± 38.5
	U	331.0 ± 3.4	2751 ± 836	720.8 ± 19.2

**Table 3.** Wood impactor test results for PLA, PAA and HIC for upright (U) and forward-flexed (F) head positions.

PPE	Head Orientation	PLA (g)	PAA (rad/s^2^)	HIC
MSA 4 Pin	F	39.6 ± 0.7	2393 ± 105	22.6 ± 0.9
	U	32.8 ± 3.0	1314 ± 24	19.9 ± 1.6
MSA 4 Ratchet	F	32.9 ± 3.1	1608 ± 106	19.3 ± 0.6
	U	28.7 ± 1.4	1395 ± 187	15.9 ± 1.2
MSA 6 Ratchet	F	28.7 ± 0.9	1831 ± 106	17.0 ± 1.4
	U	30.3 ± 1.3	1314 ± 215	20.7 ± 2.9
ERB 4 Pin	F	32.2 ± 0.8	1840 ± 199	13.9 ± 1.4
	U	34.1 ± 3.8	1509 ± 72	17.8 ± 0.7
ERB 4 Ratchet	F	32.9 ± 0.4	2192 ± 454	15.9 ± 0.7
	U	38.6 ± 3.4	1334 ± 111	19.1 ± 0.7
ERB 6 Ratchet	F	29.3 ± 0.2	2384 ± 170	15.6 ± 1.1
	U	36.0 ± 0.4	1365 ± 110	19.5 ± 0.5
Unprotected	F	231.2 ± 10.1	22841 ± 20841	461.4 ± 58.2
	U	247.1 ± 6.9	2608 ± 576	472.1 ± 11.5

**Table 4.** Lead shot impactor test results for PLA, PAA and HIC for upright (U) and forward-flexed (F) head positions.

PPE	Head Orientation	PLA (g)	PAA (rad/s^2^)	HIC
MSA 4 Pin	F	16.5 ± 0.3	1445 ± 150	5.0 ± 0.7
	U	12.7 ± 0.7	625 ± 112	2.8 ± 0.4
MSA 4 Ratchet	F	14.8 ± 1.0	1315 ± 25	4.6 ± 0.5
	U	15.1 ± 1.0	605 ± 124	4.0 ± 0.6
MSA 6 Ratchet	F	14.6 ± 0.8	1347 ± 68	4.7 ± 0.3
	U	13.2 ± 0.3	687 ± 130	3.1 ± 0.1
ERB 4 Pin	F	15.5 ± 0.1	1191 ± 144	4.3 ± 0.1
	U	12.8 ± 1.0	631 ± 63	2.9 ± 0.3
ERB 4 Ratchet	F	13.0 ± 1.5	1357 ± 97	3.1 ± 0.2
	U	13.2 ± 0.5	697 ± 115	3.0 ± 0.4
ERB 6 Ratchet	F	14.2 ± 0.7	1071 ± 104	3.9 ± 0.3
	U	14.0 ± 0.8	611 ± 55	3.3 ± 0.4
Unprotected	F	67.3 ± 14.6	1431 ± 152	17.5 ± 8.9
	U	74.5 ± 11.3	885 ± 148	25.4 ± 8.2

**Figure 4.**
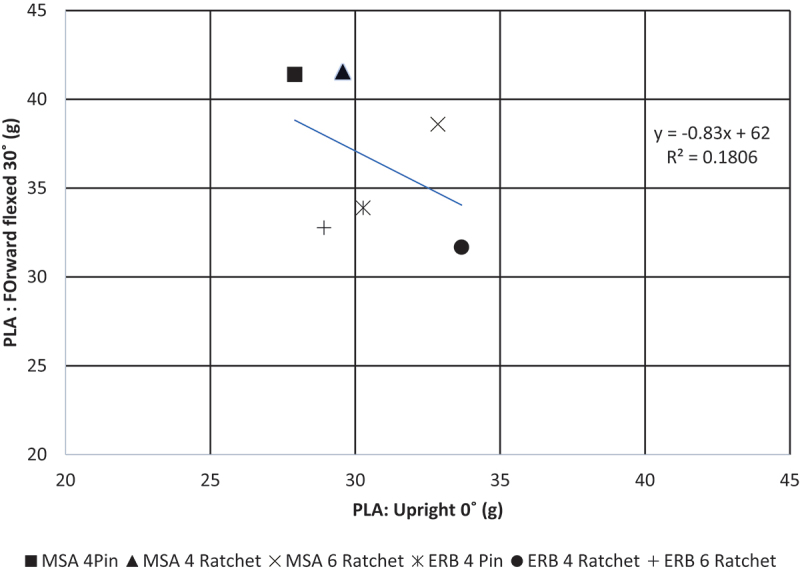
Scatter plot of PLA measurements for forward-flexed head posture versus upright head posture for a steel impactor. Note that the negative correlation (although not statistically significant), indicates that some hard hats that performed well in upright testing often performed poorly in forward-flexed conditions (and vice versa).

To investigate whether a correlation existed between protective performance of hard hats measured for upright vs. forward-flexed impacts, linear and rank correlation analyses were performed for each impactor type (steel, wood, lead shot) and outcome metric (PLA, PAA, HIC); unprotected impacts were excluded from these analyses. Correlation coefficients are provided in Table 5. None of the correlations were statistically significant (*p* > 0.16). Moreover, for both the linear and rank correlation analyses, most of the relationships with had negative correlations, indicating that hard hats that performed well in upright tests were often poor performers in forward-flexed tests (and vice versa). This is further supported by the observation that the strongest correlations (i.e. the largest magnitudes) found using both rank and linear analyses were negative. A sample scatter plot depicting such a correlation is provided in Figure 4, which shows that PLA measured from upright impacts has a negative correlation with PLA measured for the same hard hat in forward flexed impacts. In fact, for this test condition, the worst-performing helmet in upright testing was the best performer for forward-flexed testing; similarly, the best-performing helmet in upright testing was second-to-worst in forward-flexed testing.

**Table 5.** Correlation coefficients for linear and rank analyses of each helmet, comparing upright versus forward-flexed performance. Note that none of the relationships are statistically significant.

Test Condition	Injury metric	Pearson’s R	Spearman’s R
Steel	PLA	−0.425	−0.486
	PAA	−0.075	−0.486
	HIC	−0.644	−0.657
Wood	PLA	−0.003	0.143
	PAA	−0.444	−0.486
	HIC	0.053	0.257
Lead Shot	PLA	−0.308	−0.486
	PAA	0.402	0.429
	HIC	0.059	−0.257

## 4. Discussion

Fundamental epidemiological data support the fact that the most common head orientation at the time of head injuries is looking partially down (1985). However, certification standards have not been developed to account for the forward-flexed head position, and it is unclear if hard-hat manufacturers design their products for such a situation. The data set from this study is completely novel (we are aware of no studies that have ever tested hard hats on a head form with neck flexion) and its findings highlight the importance of head orientation in hard-hat performance. Comparisons between the two head orientations showed that the forward-flexed posture produced higher values for all injury metrics, with a modest increase for linear metrics and the greatest increase being seen for peak angular acceleration (7.8% for PLA, 10.9% for HIC and 41.5% for PAA). Although hard hats attenuated accelerations reasonably well for a forward-flexed head position (compared to the unprotected values), they were less effective at doing so than when the head form was upright and were least effective at attenuating angular accelerations. Moreover, for forward-flexed postures subjected to lead and steel impacts, the measured values of PAA still approached some tolerance thresholds (Schmitt 2010) even when equipped with a hard hat. Several biomechanical studies have suggested that rotational accelerations are more predictive of TBI than linear (Goldsmith and Ommaya 1984; Schmitt 2010; Kleiven 2013). The results from this study provide insight into why impacts to a forward-flexed head are prevalent in epidemiological data, but also suggest that current hard-hat designs may not be optimized for attenuating angular accelerations during impacts to a forward-flexed head.

These findings align with recent research on helmet performance under oblique impacts and non-standard head orientations (meant to simulate falls). For instance, Bottlang et al. (2022) evaluated various industrial safety helmets and found that traditional hard hats were less effective in mitigating rotational accelerations during oblique impacts compared to helmets with rotation-damping technologies. Similarly, Kim et al. (Kim et al. 2022) assessed the fall protection of Type I industrial helmets and reported that helmet performance significantly varied with impact surface conditions and helmet design features. It has also been found that chin strap usage and suspension tightness in helmet performance during non-standard impact scenarios (Wu et al. 2022). In the realm of sports, studies on bicycle helmets have demonstrated that designs incorporating features like elastomeric honeycomb structures can significantly reduce rotational head kinematics during oblique impacts (Hansen et al. 2013; Bottlang et al. 2020). These studies collectively emphasize the critical role of helmet design in protecting against rotational forces, especially during non-vertical impacts.

A fundamental finding from this present study is that the performance of individual hard hat models from upright testing conditions did not correlate with the performance of the same models in a forward-flexed condition (Figure 4). Moreover, the majority of the correlations were negative (although not significant), indicating that hard hats that performed well in upright testing often performed poorly in forward-flexed conditions (or vice versa). This suggests that the selection of a hard hat based on results from upright test conditions may in fact result in reduced protection during the most common and injurious accident scenarios involving flexed postures. Future studies should seek to identify mechanistic sources of these negative correlations (i.e. design features that cause helmets to perform well in either vertical or forward-flexed conditions) such that new generations of hard hats can be developed that respond well to impacts under a variety of head postures.

While it was not a specific goal of this study to compare brands or design features relative to protective performance, the inconsistency in performance relative to head orientation and the difference in relative performance when comparing only the forward flexed results for the two brands of hard hats, suggests a design factor is influencing head acceleration attenuation. For example, all ERB models performed significantly better than all the MSA models in terms of HIC (*p* < 0.05). Also, the ERB models generally reduced PLA and PAA more effectively than the MSA models (although the differences were not statistically significant). Even though models were selected from each manufacturer based on their similarity in features (a 4-point pin-lock and ratcheting model as well as a 6-point ratcheting model were used from each), this difference suggests that some other design feature on the ERB models is causing this improvement.

**Figure 5.**
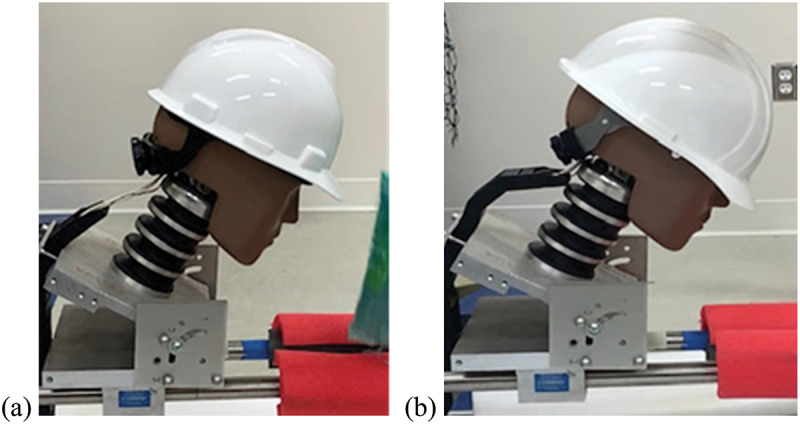
Still frames taken from high-speed video showing (a) an MSA hard hat and (b) an ERB hard hat immediately post impact.

The analysis of the high-speed video footage of impacts revealed that the ERB models displaced more and tended to decouple from the head form, which provides a potential mechanistic explanation for the greater attenuation of accelerations experienced during testing. This physical phenomenon can be observed in the still frames shown in Figure 5, where the change in position of the ERB suspension system is evident (the brim has tilted to the level of the nose, and the backstrap of the headband has fallen beneath the occiput), while the MSA model has remained in approximately the same position as before impact. As seen in Figure 2, the headband, ratchet contact materials, and the connecting pin from the headband to the back section of the tightening system of the ERB models are substantially different when compared to the MSA models. Qualitatively, the ERB headband and tightening mechanism materials seemed to be made from materials with less friction than the MSA models, potentially enabling the hard hat to move independently of (i.e. decoupled from) the head form. Thus, as previously mentioned in literature, the decoupling effect is hypothesized to attenuate head acceleration (Hansen et al. 2013; Taylor 2018; Strickland and Bevill 2019).

Some limitations apply to this study that warrant discussion. First, as is the case with all ATD testing, the Hybrid III used in this study is not perfectly bio-fidelic – there are differences in material properties of the Hybrid III compared to the actual human head, notably including that the ATD neck is stiffer than a human neck (particularly for axial loading). Another limitation to this study is that only six hard hat models were tested. While this is relatively large in comparison to many prior studies that have experimentally tested hard hats, there are nevertheless numerous models of hard hats commercially available. It is not possible to test all hard hat designs currently on the market. The hard hats that were selected were chosen since they are amongst the best-selling models and their features are generally representative of most commercial models. The general features that were identified as being potentially important to performance in this study can also be seen or improved by most of the other models. However, given the substantial performance differences across brands, which were potentially attributable to relatively subtle design differences, it is possible that other hard hat models/brands that were not evaluated as part of this study could exhibit different performance behavior or trends.

Taken together, this study provides novel data regarding the performance of hard hats in a forward-flexed head orientation, in contrast to the upright posture used in standard certification testing. The findings offer compelling evidence that construction PPE should be evaluated under a wider range of head postures to better reflect real-world injury scenarios and improve worker safety. Notably, hard hats did not attenuate accelerations as effectively when the head was forward-flexed, raising questions about their protective capacity during common work activities that involve neck flexion. To address these concerns, current certification standards – such as ANSI Z89.1 and equivalent international protocols – should be revised to include angled or non-neutral impact configurations. Incorporating such conditions into standardized testing would better align with epidemiological data and workplace biomechanics, ultimately improving the relevance of certification outcomes. Moreover, updating these standards would provide clear incentive for manufacturers to innovate on critical aspects of helmet design, such as strap suspension systems, energy-absorbing liners and outer shell geometries, to enhance performance in a broader range of impact orientations. Future research should extend these findings by evaluating hard hat performance under additional real-world impact conditions, including oblique (or offset) impacts and a wider spectrum of head and body postures. These efforts will be essential to developing PPE that better protects workers from the full range of threats encountered on construction sites.
